# Exploring the underlying molecular mechanism of liver cancer cells under hypoxia based on RNA sequencing

**DOI:** 10.1186/s12863-022-01055-9

**Published:** 2022-05-19

**Authors:** Xin Zhao, Wenpeng Liu, Baowang Liu, Qiang Zeng, Ziqiang Cui, Yang Wang, Jinglin Cao, Qingjun Gao, Caiyan Zhao, Jian Dou

**Affiliations:** 1 Department of Hepatobiliary Surgery, The Third Hospital of Hebei Medical University, No.139 Ziqiang Road, Shijiazhuang City, 050051 Hebei Province China; 2 Department of Infectious Disease, The Third Hospital of Hebei Medical University, Shijiazhuang, China

**Keywords:** Liver cancer, Hypoxia, RNA sequencing, MicroRNAs

## Abstract

**Background:**

The aim of our study was to use the differentially expressed mRNAs (DEmRNAs) and differentially expressed miRNAs (DEmiRNAs) to illustrate the underlying mechanism of hypoxia in liver cancer.

**Methods:**

In this study, a cell model of hypoxia was established, and autophagy activity was measured with western blotting and transmission electron microscopy. The effect of hypoxia conditions on the invasion of liver cancer cell was evaluated. RNA sequencing was used to identify DEmRNAs and DEmiRNAs to explore the mechanism of hypoxia in liver cancer cells.

**Results:**

We found that autophagy activation was triggered by hypoxia stress and hypoxia might promote liver cancer cell invasion. In addition, a total of 407 shared DEmRNAs and 57 shared DEmiRNAs were identified in both HCCLM3 hypoxia group and SMMC-7721 hypoxia group compared with control group. Furthermore, 278 DEmRNAs and 24 DEmiRNAs were identified as cancer hypoxia-specific DEmRNAs and DEmiRNAs. Finally, we obtained 19 DEmiRNAs with high degree based on the DEmiRNA-DEmRNA interaction network. Among them, hsa-miR-483-5p, hsa-miR-4739, hsa-miR-214-3p and hsa-miR-296-5p may be potential gene signatures related to liver cancer hypoxia.

**Conclusions:**

Our study may help to understand the potential molecular mechanism of hypoxia in liver cancer.

**Supplementary Information:**

The online version contains supplementary material available at 10.1186/s12863-022-01055-9.

## Introduction

Liver cancer is the sixth most commonly diagnosed cancer and the third leading cause of cancer death worldwide in 2020, a great challenge for public health due to its high morbidity and high mortality [1]. In China, more than 466,100 people have been diagnosed with liver cancer, and about 422,100 people die of liver cancer each year [2]. At present, liver transplantation, surgical resection and ablation are the main curative therapy ways for early hepatocellular carcinoma [3]. Although some progress in uncovering the pathogenesis of liver cancer, there remains difficulties in early diagnosis due to the lack of effective detection. In addition, most of liver cancer is diagnosed at advanced stages due to its high aggressiveness and rapid proliferation [3, 4]. Thus, it is urgent to understand the deep molecular mechanisms for liver cancer metastasis and discover novel targets for the detection of early liver cancer.

MicroRNAs (miRNAs) are a class of small RNAs involved in the post-transcriptional regulation of many target genes that may participate in tumor formation as oncogenes and tumor suppressor genes [5]. Increasing studies have revealed that dysregulated miRNAs may be involved in the initiation, development and prognosis of malignant tumors including liver cancer [6–8]. Currently, high-throughput combined with bioinformatics methods has been applied to reveal the pathogenesis of liver cancer progression and to identify novel biomarkers associated with diagnosis and prognosis of liver cancer [9, 10].

Hypoxia is one of the major features of cancer, affecting gene expression, angiogenesis, cell proliferation, cell invasion and related processes of tumor biology [11–13]. Previous studies have found that hypoxia can cause autophagy, which plays an important role in the progression of cancer [14]. Autophagy has been shown to play a central role in the formation, growth, invasion, and migration of tumors, and play a dual role in multiple malignancies, either as a tumor promoter or as a tumor suppressor [15, 16]. Impaired autophagy through deletion of Beclin-1, ATG5 or ATG7 in mice promotes spontaneous liver tumorigenesis in aged mice [17]. Song et al. found that autophagy is a protective mechanism involved in the resistance to chemotherapy under hypoxic conditions in liver cancer [18]. In addition, a growing number of studies have shown a relationship between tumor hypoxia characteristics and tumor immunosuppression and immune escape [19, 20]. Tumor hypoxia is also considered as an effective target for cancer treatment [21]. However, the function of hypoxia in the development of liver cancer and its underlying mechanism are still not fully understood. Therefore, exploring the molecular mechanism of liver cancer hypoxia is conducive to the discovery of new tumor treatment strategies.

In this study, the method of hypoxia induced cells was used to explore the biological function of liver cancer cells under hypoxia. HCCLM3, SMMC-7721 and LX2 cell lines were treated under hypoxic (hypoxia group) or normoxic (control group) conditions for RNA sequencing. Then, the differentially expressed mRNAs (DEmRNAs) and miRNAs (DEmiRNAs) between the hypoxia group and control group were obtained. In addition, the liver cancer cell hypoxia-specific DEmRNAs and DEmiRNAs were also identified. The DEmiRNA-DEmRNA interaction network and functional enrichment analysis of DEmRNAs targeted with DEmiRNAs were used to study the underlying mechanism of hypoxia in liver cancer cells. Our data provides a new perspective for revealing the role and its related molecular mechanism of hypoxia in liver cancer.

## Material and methods

### Cell culture

Liver cancer cell lines (SMMC-7721, HepG2, HCCLM3 and MHCC97H) and human hepatic cell line LX2 were purchased from the American Type Culture Collection. Cells were cultured in DMEM (Gibco, Waltham, MA, USA) containing 10% fetal bovine serum (Gibco) and 1% penicillin/streptomycin at 37°C with 5% CO_2_, and a humidified atmosphere, considered as the normoxic conditions.

### Experimental design

Cell lines cultured in complete medium were served as control. The cells were seeded in 6-well plates overnight, subsequently incubated in hypoxia incub ator (Sanyo Electric Co., Ltd., Osaka, Japan) containing humidified hypoxic air (1% O_2_, 5% CO_2_, and 94% N_2_) at 37°C for 12h. Control cells were incubated under normoxic conditions. The cells were divided into normal culture group and hypoxia group.

### Electron microscopy

After indicated treatments, the cells were harvested and fixed with 2.5% glutaraldehyde at 4°C overnight. The samples were suspended in PBS with 1% osmic acid. After dehydration and embedding, the 70-nm-thick sections were prepared on uncoated copper grids with an Ultrotome (Leica Microsystems, Wetzlar, Germany) and double-stained with uranyl acetate and lead citrate for 15 min at room temperature. Autophagosomes were observed under JEM 1230 transmission electron microscope (JEOL, Japan).

### Western blotting analysis

Subsequent to the indicated treatments, protein of cells was harvested with RIPA buffer (Beyotime, Shanghai, China) supplemented with PMSF (Beyotime) and determined using a bicinchoninic acid assay kit (Beyotime). Proteins were separated with 10% or 12% SDS-PAGE gels and transferred PVDF membranes (Merck Millipore, Billerica, MA, USA), which were blocked with 5% non-fat milk for 1 h at room temperature. The membranes were incubated with primary antibody at 4°C overnight and then blotted with secondary antibody for 2 h at room temperature. The bands were detected using enhanced chemiluminescence (Merck Millipore). The primary antibodies against p62 (1:1000), LC3 (1:1000), and β-actin (1:1000) were obtained from Cell Signaling Technology (Danvers, MA, USA).

### Transwell assay

Cell invasion ability was measured with Transwell assays (Merck Millipore). Cells were re-suspended in 100 μL serum-free medium at a density of 1 × 10^4^, and then inoculated into the upper chambers coated with Matrigel (BD Bioscience, Franklin Lakes, NJ). Whereas, DMEM medium embracing 10% FBS was added to the lower chamber. At 24 h post incubation, the invasive cells were fixed with 4% paraformaldehyde and dyed with 0.5% crystal violet for 20 min, and counted under a light microscope (Olympus Tokyo, Japan) in five random fields.

### Statistical Analysis

All experimental data were presented as the mean±standard deviation, and each experiment was performed at least three times. The statistical analyses were performed by student’s t-test or one-way ANOVA using SPSS version 22.0 (IBM Corp, Armonk, NY, USA). A *p*-values < 0.05 was denoted statistical significance.

### RNA isolation and sequencing

HCCLM3, SMMC-7721 and LX2 cell lines were used as the research objects, which were treated under hypoxic (hypoxia group) or normoxic (control group) conditions. Total RNA was extracted from cells using TRIzol reagent (Life Technologies, CA, USA) according to the manufacturer’s protocol. Spectrophotometric and agarose gel electrophoresis was used to evaluate the quality and quantity of total RNA. Illumina Hiseq Xten platform (Illumina, San Diego, CA, USA) was performed to conduct sequencing of mRNA. Sequencing of miRNA was carried out using BGIseq-500 platform (BGI, China). Significantly DEmRNAs and DEmiRNAs were defined using edgeR v 3.24 (http://www.bioconductor.org/packages/release/bioc/html/edgeR.html) with a threshold of |log_2_FC|>1 and *p*-value<0.05. The volcano maps of the DEmRNAs and DEmiRNAs were produced using R package. Venny 2.1.0 (http://bioinfogp.cnb.csic.es/tools/venny/) was applied to acquire the shared and specific DEmRNAs and DEmiRNAs.

### DEmiRNA-DEmRNA interaction analysis

We used the miRWalk 3.0 (http://mirwalk.umm.uni-heidelberg.de/) to search for the target genes of liver cancer hypoxia-specific DEmiRNA. Three bioinformatic algorithms (TargetScan, miRDB, and miRTarBase) were utilized to predict the supposed target DEmRNAs of DEmiRNAs. In addition, the DEmiRNA-DEmRNA pairs recorded by≥1 algorithms in which DEmRNA was negatively correlated with DEmiRNAs were retained for further investigation. The DEmiRNA-DEmRNA interaction networks were constructed by using Cytoscape 3.7.1 (http://www.cytoscape.org/).

### Functional enrichment

We further explored the main biological functions of the identified DEmRNAs targeted with DEmiRNAs via the Gene Ontology (GO) and Kyoto Encyclopedia of Genes and Genomes (KEGG) pathway enrichment analysis. David 6.8 (https://david.ncifcrf.gov/) was used to carried out the GO and KEGG pathway enrichment analysis. A *p*-value <0.05 as the cut-off value was deemed statistically significant.

## Results

### Hypoxia induced autophagy activation in liver cancer cells

**Fig. 1:**
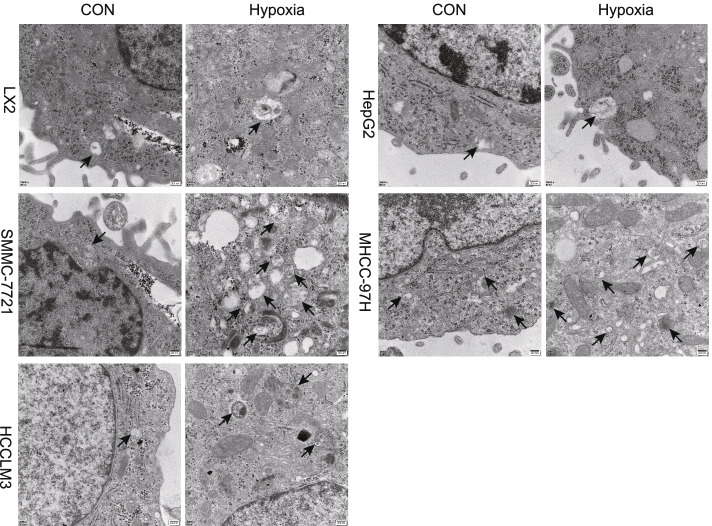
Effects of hypoxia on autophagy in liver cancer cells. Autophagic vesicles were detected by electron microscopy. The arrows designate the autophagic vesicles.

**Fig. 2:**
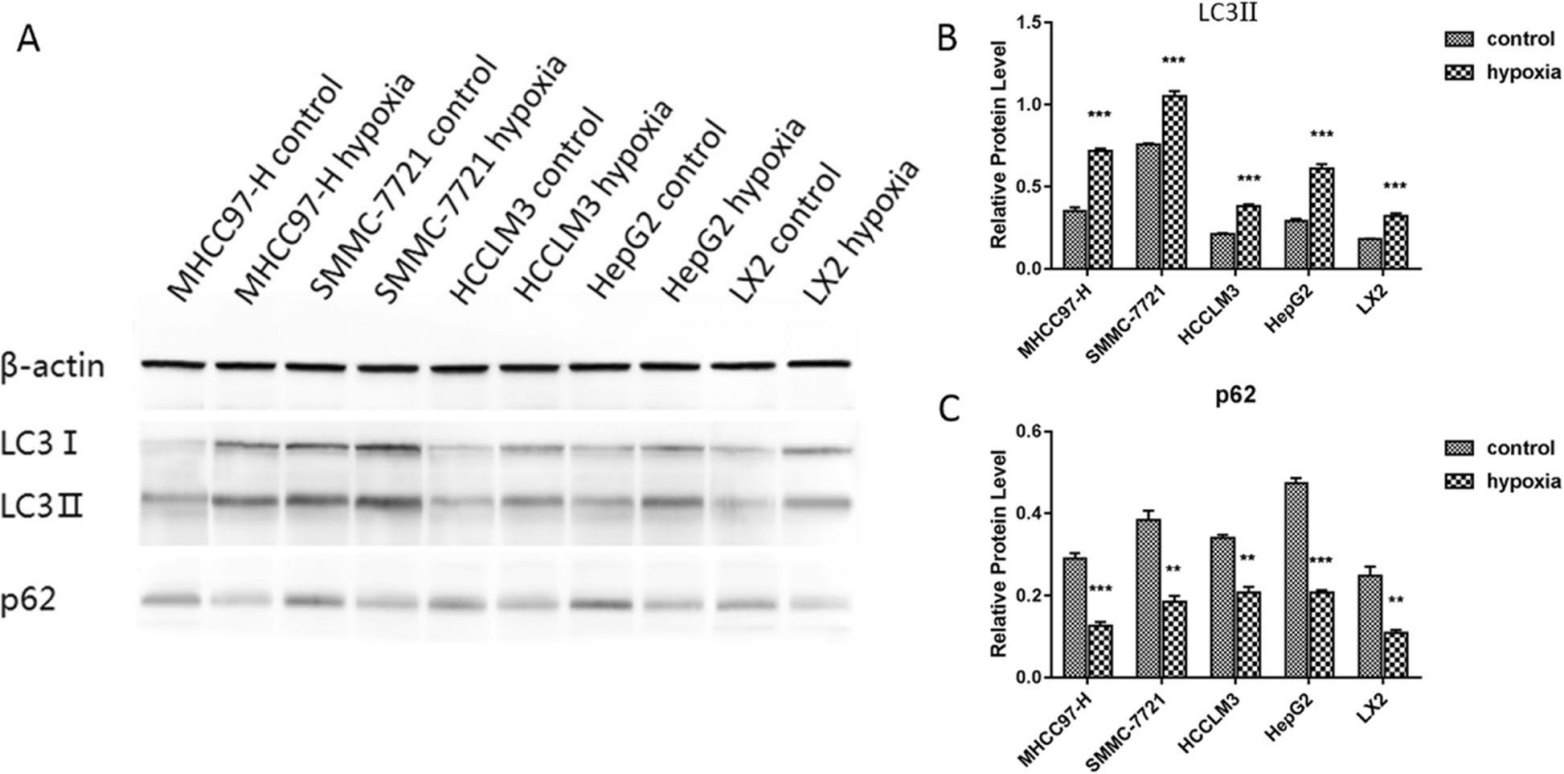
Effects of hypoxia on autophagy-related proteins. The indicated proteins were examined using western blot analysis. β-actin was detected as the loading control. **p-value < 0.01, ***p-value < 0.001

**Fig. 3:**
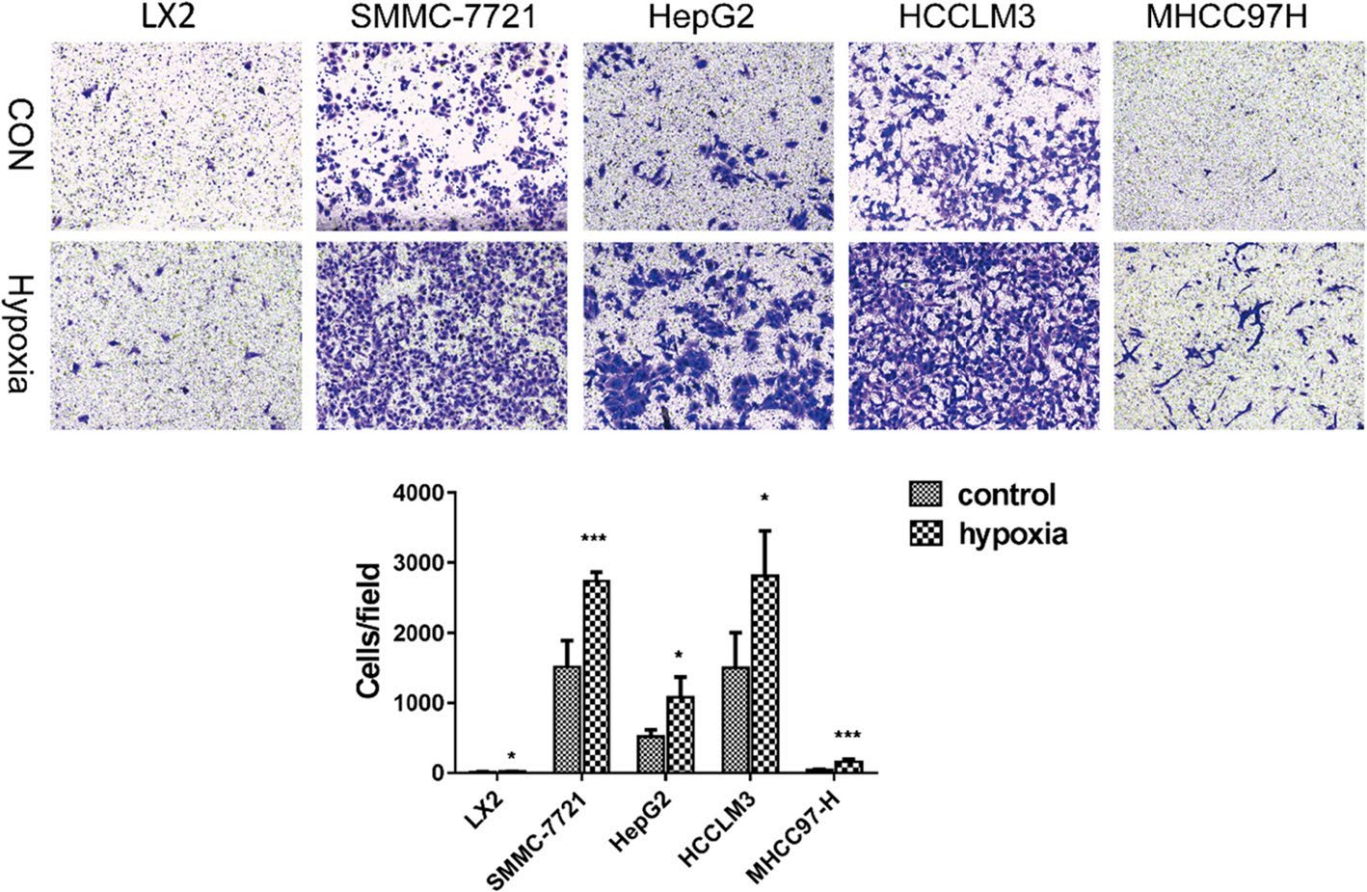
Effects of hypoxia on invasion of liver cancer cells. Transwell assay found that hypoxia induced the invasion of liver cancer cells. *p-value < 0.05, **p-value < 0.01

To explore whether autophagy activation was triggered by hypoxia stress in liver cancer cells, we detected autophagic vesicles in SMMC-7721, HepG2, HCCLM3, MHCC97H and LX2 cell lines by electron microscopy. The number of autophagic vesicles was significantly increased under hypoxic conditions (Fig. 1). Then, the protein expression levels of LC3II and p62, which are considered reliable indicators of autophagy, were examined. The western blotting results demonstrated that the autophagy activation was triggered by hypoxia conditions. The expression level of LC3II was increased in hypoxic conditions group, while p62 protein expression was significantly decreased (Fig. 2). Taken together, these results indicated that hypoxia induced autophagy in liver cancer cells. Further, we studied the effect of hypoxia conditions on the invasion of liver cancer cell. The transwell assay results indicated that hypoxia resulted in significantly increased cell invasion in liver cancer cells (Fig. 3). In summary, our results demonstrated that hypoxia might promote liver cancer cell invasion.

### Identification of DEmRNAs and DEmiRNA

**Fig. 4:**
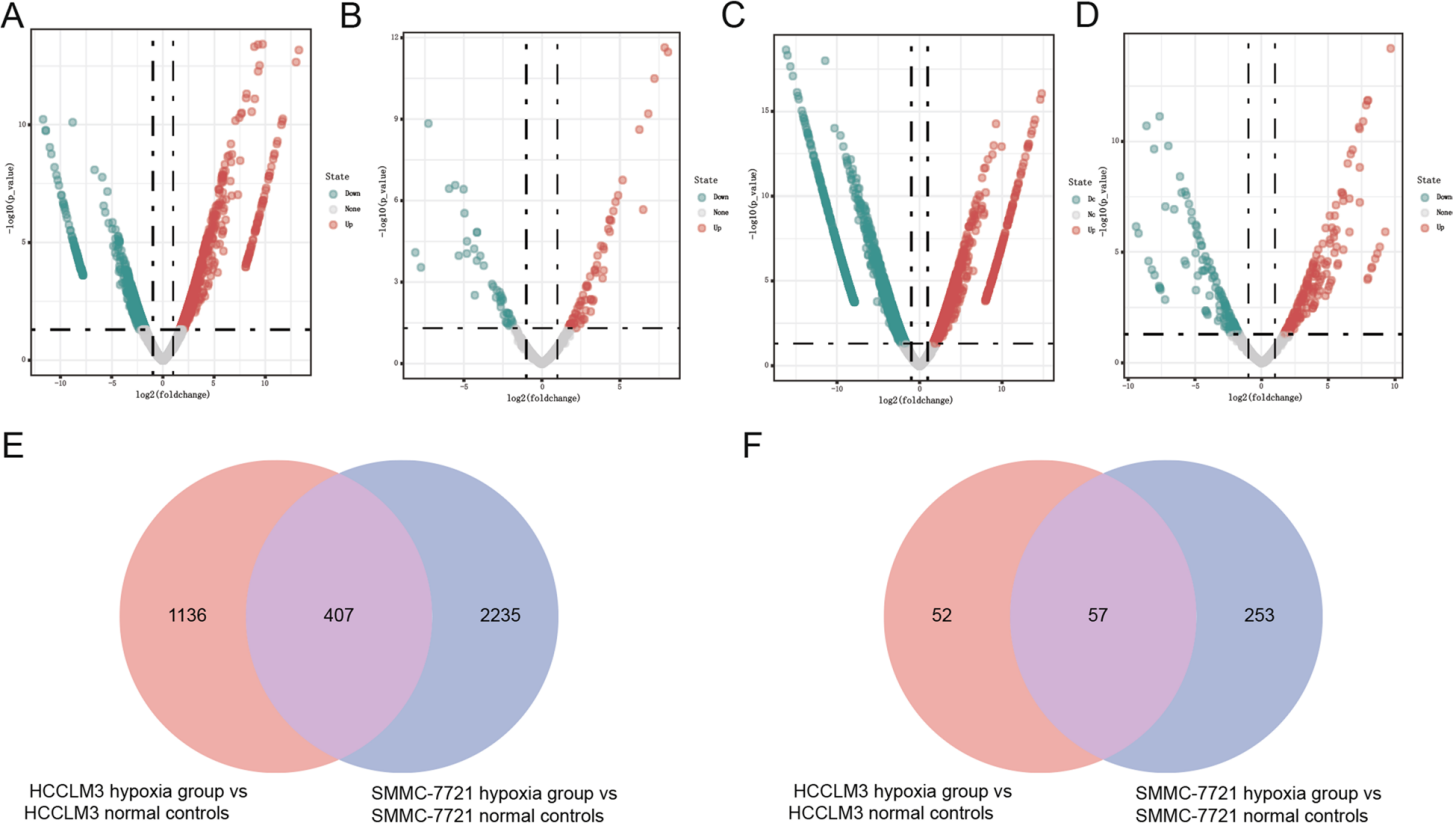
DEmRNA and DEmiRNA in liver cancer cells hypoxia group vs. control group. (**A**) The volcano plot of DEmRNAs in HCCLM3 hypoxia group vs. HCCLM3 normal controls. (**B**) The volcano plot of DEmRNAs in SMMC-7721 hypoxia group vs. SMMC-7721 normal controls. (**C**) The volcano plot of DEmiRNAs in HCCLM3 hypoxia group vs. HCCLM3 normal controls. (**D**) The volcano plot of DEmiRNAs in SMMC-7721 hypoxia group vs. SMMC-7721 normal controls. (**E**) Venn diagram of shared DEmRNAs in both HCCLM3 hypoxia group and SMMC-7721 hypoxia group compared with normal controls. (**F**) Venn diagram of shared DEmiRNAs in both HCCLM3 hypoxia group and SMMC-7721 hypoxia group compared with normal controls

**Fig. 5:**
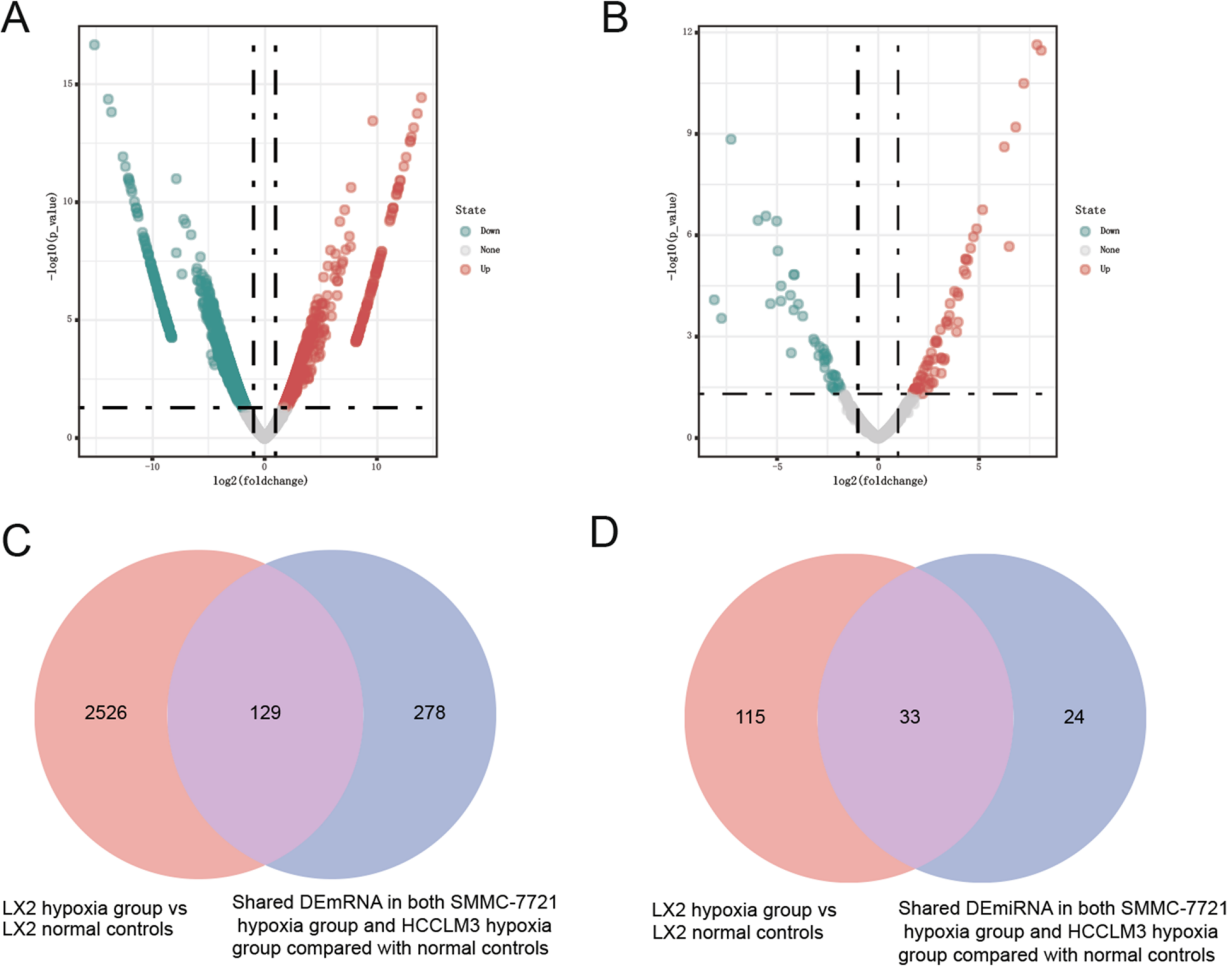
DEmRNA and DEmiRNA in LX2 hypoxia group vs. LX2 control group. (**A**) The volcano plot of DEmRNAs in LX2 hypoxia group vs. LX2 normal controls. (**B**) The volcano plot of DEmiRNAs in LX2 hypoxia group vs. LX2 normal controls. (**C**) Venn diagram of liver cancer hypoxia-specific DEmRNAs. (**D**) Venn diagram of liver cancer hypoxia-specific DEmRNAs

The raw-data has been uploaded to Gene Expression Omnibus (GEO) (GSE185971; https://www.ncbi.nlm.nih.gov/geo/query/acc.cgi?acc=GSE185971) database. A total of 1543 DEmRNAs (844 up-regulated and 699 down-regulated DEmRNAs) and 109 DEmiRNAs (67 up-regulated and 42 down-regulated DEmiRNAs) were identified in HCCLM3 hypoxia group vs. HCCLM3 control group. Volcano plots of the DEmRNAs and DEmiRNAs in HCCLM3 hypoxia group vs. HCCLM3 control group were shown in Fig. 4A and C, respectively. We obtained 2642 DEmRNAs (1153 up-regulated and 1489 down-regulated DEmRNAs) and 310 DEmiRNAs (188 up-regulated and 122 down-regulated DEmiRNAs) in SMMC-7721 hypoxia group vs. SMMC-7721 control group. Volcano plots of the DEmRNAs and DEmiRNAs in SMMC-7721 hypoxia group vs. SMMC-7721 control group were shown in Fig. 4B and D, respectively. In addition, a total of 407 shared DEmRNAs and 57 shared DEmiRNAs were identified in both HCCLM3 hypoxia group and SMMC-7721 hypoxia group compared with control group (Fig. 4E and F). A total of 2625 DEmRNAs (1035 up-regulated and 1620 down-regulated DEmRNAs) and 148 DEmiRNAs (82 up-regulated and 66 down-regulated DEmiRNAs) were identified in LX2 hypoxia group vs. LX2 control group. Volcano plots of the DEmRNAs and DEmiRNAs in LX2 hypoxia group vs. LX2 control group were displayed in Fig. 5A and B, respectively. Moreover, 278 DEmRNAs were identified as liver cancer hypoxia-specific DEmRNAs and 24 DEmiRNAs were identified as liver cancer hypoxia-specific DEmiRNAs (Fig. 5C and D).

### DEmiRNA-DEmRNA interaction analysis

**Fig. 6:**
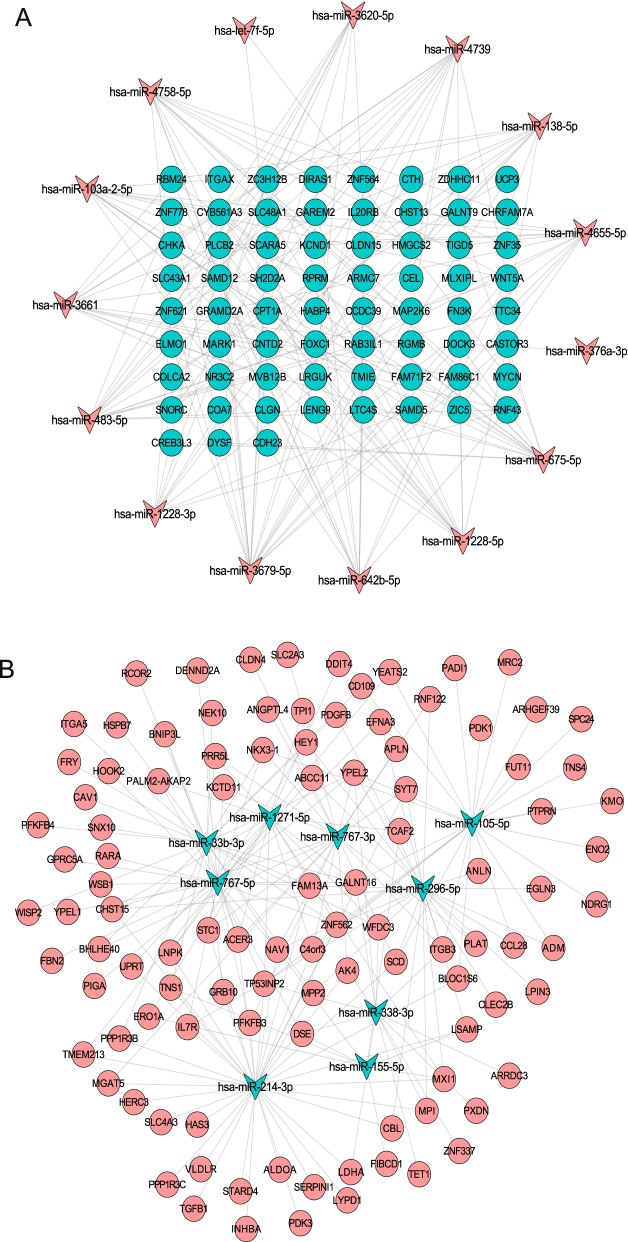
DEmiRNA-DEmRNA interaction network. (**A**) up-regulated DEmiRNA. (B) down-regulated DEmiRNA. The inverted triangles and ellipses were represented the DEmiRNAs and DEmRNA, respectively. Red and green color represented up- and down-regulation, respectively

Next, liver cancer hypoxia-specific DEmiRNA-DEmRNA interaction network was constructed for up-regulated and down-regulated miRNAs, respectively. For the up-regulated DEmiRNAs, a total of 175 DEmiRNA-DEmRNA pairs, including 15 DEmiRNAs and 67 DEmRNAs were identified, and the DElncRNA-DEmRNA interaction network was consisted of 82 nodes and 175 edges (Fig. 6A). For the down-regulated DEmiRNAs, a total of 203 DEmiRNA-DEmRNA pairs, including 9 DEmiRNAs and 105 DEmRNAs were identified, and the DElncRNA-DEmRNA interaction network was consisted of 114 nodes and 203 edges (Fig. 6B). Hsa-miR-3679-5p (degree=20), hsa-miR-483-5p (degree=15), hsa-miR-675-5p (degree=15), hsa-miR-642b-5p (degree=14), hsa-miR-4739 (degree=14), hsa-miR-1228-5p (degree=14), hsa-miR-3661 (degree=13), hsa-miR-4758-5p (degree=13), hsa-miR-103a-2-5p (degree=12) and hsa-miR-4655-5p (degree=12) were top 10 up-regulated DEmiRNAs with high degree. All down-regulated DEmiRNAs with high degree included hsa-miR-214-3p (degree=36), hsa-miR-767-5p (degree=28), hsa-miR-33b-3p (degree=26), hsa-miR-296-5p (degree=25), hsa-miR-105-5p (degree=24), hsa-miR-767-3p (degree=22), hsa-miR-1271-5p (degree=18), hsa-miR-338-3p (degree=13) and hsa-miR-155-5p (degree=8).

### Functional enrichment analysis of liver cancer hypoxia-specific DEmRNAs

**Fig. 7:**
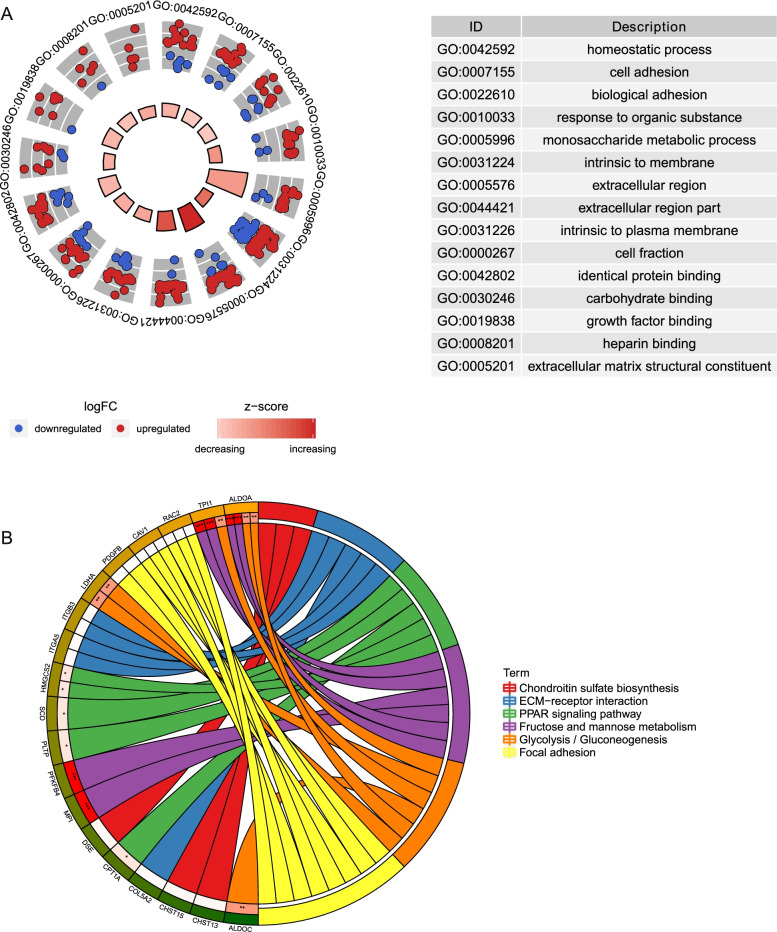
Functional enrichment analysis of DEmRNAs. (A) GO enrichment analyses of DEmRNAs (B) KEGG pathway enrichment analyses of DEmRNAs

As displayed in Fig. 7A, GO analysis found that in the biological process, DEmRNAs targeted with DEmiRNAs mainly enriched in regulation of cell proliferation and negative regulation of cell proliferation. In the cellular component analysis, DEmRNAs were primarily enriched in intrinsic to membrane and plasma membrane part. Molecular function analysis indicated that DEmRNAs were mainly enriched in identical protein binding and 6-phosphofructo-2-kinase activity. KEGG pathway enrichment analysis indicated that DEmRNAs targeted with DEmiRNAs were significantly enriched in fructose and mannose metabolism, chondroitin sulfate biosynthesis, glycolysis/gluconeogenesis and PPAR signaling pathway (Fig. 7B) [22–24].

## Discussion

Liver cancer is a common malignant tumor that is considered to be one of the leading cause of cancer-related deaths worldwide due to the lack of effective treatment [25]. Hypoxia is a key regulator in liver cancer progression [26]. Therefore, it is urgent to elucidate the pathogenesis and seek hypoxia -associated therapeutic targets of liver cancer.

In this study, we found that the autophagy activation can be triggered by hypoxia conditions as evidenced by increased autophagic vesicles formation, increased LC3II level and decreased p63 level. In addition, hypoxia might promote liver cancer cell invasion. The bioinformatics analysis identified 407 shared DEmRNAs and 57 shared DEmiRNAs in both HCCLM3 hypoxia group and SMMC-7721 hypoxia group compared with control group. Furthermore, 278 DEmRNAs and 24 DEmiRNAs were identified as cancer hypoxia-specific DEmRNAs and DEmiRNAs. Finally, we performed the DEmiRNA-DEmRNA interaction network and functional enrichment analysis of DEmRNAs targeted with DEmiRNAs to uncover the underlying mechanism of hypoxia in liver cancer cells. In DEmiRNA-DEmRNA interaction network, we found 10 up-regulated DEmiRNAs and 9 down-regulated DEmiRNAs with high degree.

Recently, hsa-miR-483-5p has been reported to be involved in the progression of multiple malignancies. For instances, hsa-miR-483-5p is markedly reduced in gliomas, and overexpression of miR-483-5p suppressed glioma cell proliferation and induced a G0/G1 arrest, whereas miR-483-5p inhibition promoted cell proliferation, suggesting that hsa-miR-483-5p serves as a tumor suppressor [27]. Hsa-miR-483-5p has been demonstrated to be significantly overexpressed in patients with adrenocortical carcinoma, and is considered as a minimally invasive marker for preoperative malignant tumor [28]. Hsa-miR-483-5p was significantly up-regulated in liver cancer patients than in liver cirrhosis patients, and it is considered as a non-invasive biomarker for the diagnosis of liver cancer due to its good diagnostic value [29]. It has been reported that miR-483-5p is associated with poor prognosis of hepatocellular carcinoma [30]. A study reported that hsa-miR-483-5p promotes hepatocellular carcinoma cell migration and invasion in vitro and increases intrahepatic metastasis in nude mice [31]. In this study, hsa-miR-483-5p was defined as liver cancer hypoxia-specific DEmRNA, which was significantly up-regulated in liver cancer cells. Besides, hsa-miR-483-5p was one of DEmiRNAs with high degree in DEmiRNA-DEmRNA interaction network, indicating that this miRNA may be involved in the pathologic mechanism of liver cancer. Thence, the role of hsa-miR-483-5p on hypoxia in liver cancer cells needs to be further clarified in future.

In our study, hsa-miR-4739 was identified as liver cancer hypoxia-specific DEmRNA and increased in liver cancer cells. In addition, hsa-miR-4739 was one of DEmiRNAs with high degree in DEmiRNA-DEmRNA interaction network. However, there is no directive evidence to support the involvement of hsa-miR-4739 in liver cancer. Although the function of hsa-miR-4739 on the progression of liver cancer has not been studied, available evidence shows that hsa-miR-4739 plays significant roles in other cancers [32, 33]. Silencing β-catenin expression can inhibit the proliferation of gastric cancer cells, promote cell apoptosis, and weaken the invasion ability of gastric cancer, accompanied by the increase of hsa-miR-4739, which indicates that hsa-miR-4739 may be involved in the occurrence and progression of gastric cancer [32]. Hsa-miR-4739 is significantly reduced in prostate cancer and is involved in the occurrence and progression of prostate cancer [33]. The role of hsa-miR-4739 in liver cancerwill be further revealed.

Hsa-miR-214-3p, located at the chromosomal region 1q24.3, is an mRNA involved in the occurrence, growth and development of cancer [34]. A previous study had reported that hsa-miR-214-3p is reduced in endometrial cancer tissues and its overexpression decreases the proliferation, migration, and invasion of endometrial cancer cells [35]. Notably, hsa-miR-214-3p has been found to decrease in liver cancer tissues and is closely associated with fibrotic stages [36, 37]. Recent research reported that hsa-miR-214-3p is decreased in liver cancer tissues and its overexpression hinders cell proliferation, cell cycle arrest at G1 phase, and induces cell apoptosis in liver cancer cells [38]. It has been reported that hsa_circ_0008450 inhibits the progression of liver cancer by sponging hsa-miR-214-3p to promote the expression of EZH2 protein [39]. Other study has demonstrated that lncRNA HCG18 promotes the proliferation and migration of liver cancer by hsa-miR-214-3p to regulate the expression of CENPM protein [40]. LINC00665 accelerated cell growth and Warburg effect through sponging miR-214-3p to increase MAPK1 expression in hepatocellular carcinoma [41]. Our results indicated that hsa-miR-214-3p expression was down-regulated in liver cancer cell lines, which was consistent with previous reports. Hsa-miR-214-3p was identified as liver cancer hypoxia-specific DEmRNA, suggesting that hsa-miR-214-3p may be involved in the hypoxia of liver cancer cells.

Up to date, hsa-miR-296-5p has been shown to act as a tumor suppressor to modulate cell processes by regulating targeted genes or downstream signaling pathway in a variety of cancers. For example, hsa-miR-296-5p represses non-small cell lung cancer progression via directly targeting PLK1 [42]. Hsa-miR-296-5p negatively regulates STAT3 signaling and can function as a tumor suppressor to depress cell metastasis of esophageal squamous cell carcinoma [43]. Hsa-miR-296-5p suppresses the epithelial-mesenchymal transition process of liver cancer via regulating NRG1 expression through cell-autonomous mechanism [44]. In addition, miR-296-5p exerts an inhibitory effect on stemness potency of hepatocellular carcinoma cells via Brg1/Sall4 axis [45]. Hsa-miR-296-5p is reduced in liver cancer tissues and cell lines and its overexpression inhibited liver cancer progression via directly targeting CNN2 [46]. Herein, hsa-miR-296-5p was identified as a liver cancer hypoxia-specific DEmRNA and it may be involved in the hypoxia of liver cancer cells autophagy. However, the detailed role of hsa-miR-296-5p in the development of liver cancer still needs to be elaborated.

In summary, autophagy activation under hypoxia conditions was proven in this study and the potential hypoxia-associated targets were identified based on the RNA sequencing and bioinformatics analysis. Specifically, hsa-miR-483-5p, hsa-miR-4739, hsa-miR-214-3p and hsa-miR-296-5p were considered as potential gene signatures related to liver cancer hypoxia. This work may provide a scientific evidence about the molecular mechanism of liver cancer. Although we have identified multiple novel DEmiRNAs associated with liver cancer hypoxia, additional experiments at higher molecular levels such as downregulation of some genes and other important regulators will be performed to reveal more precise mechanisms of hypoxia in liver cancer. Also, using more advanced techniques to detect autophagy such as immunofluorescence is recommended.

### Clinical significance

Hypoxia is a key regulator in liver cancer progression. Understanding the molecular mechanism of hypoxia- in liver cancer cells is beneficial to explore new tumor treatment options. In this study, we used the method of hypoxia treatment to explore the biological function of liver cancer cells under hypoxia. In addition, the liver cancer cell hypoxia-specific DEmRNAs and DEmiRNAs were also identified. This study provides potential clinical biomarkers for the early diagnosis and management of liver cancer. In addition, the identification of biomarkers of liver cancer may help to understand the molecular mechanism of hypoxia. The next important step is to expand the sample size to study their specific molecular mechanisms to help clinical practice.

## Supplementary Information

**Additional file1:.** Figure S1. The original images of western blot.

## Data Availability

The raw-data has been uploaded to Gene Expression Omnibus (GEO) (GSE185971; https://www.ncbi.nlm.nih.gov/geo/query/acc.cgi?acc=GSE185971).
