# Supplementary figures and images for: Exploring the underlying molecular mechanism of liver cancer cells under hypoxia based on RNA sequencing
Source: BMC Genom Data. 2022 May 19;23:38. doi: 10.1186/s12863-022-01055-9 (PMC9121577; doi:10.1186/s12863-022-01055-9)

**Figure S1** The original images of western blot.

β-actin


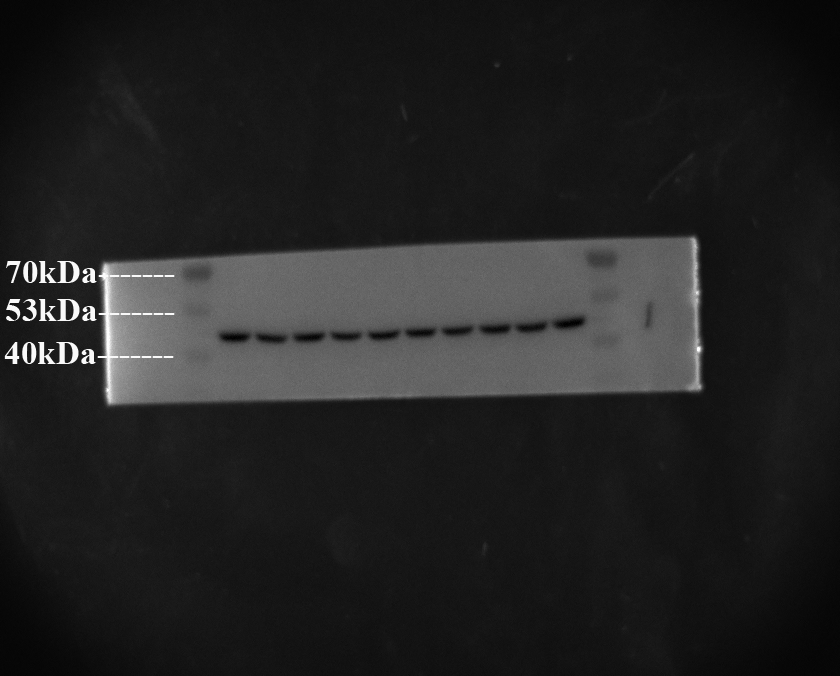


LC3II


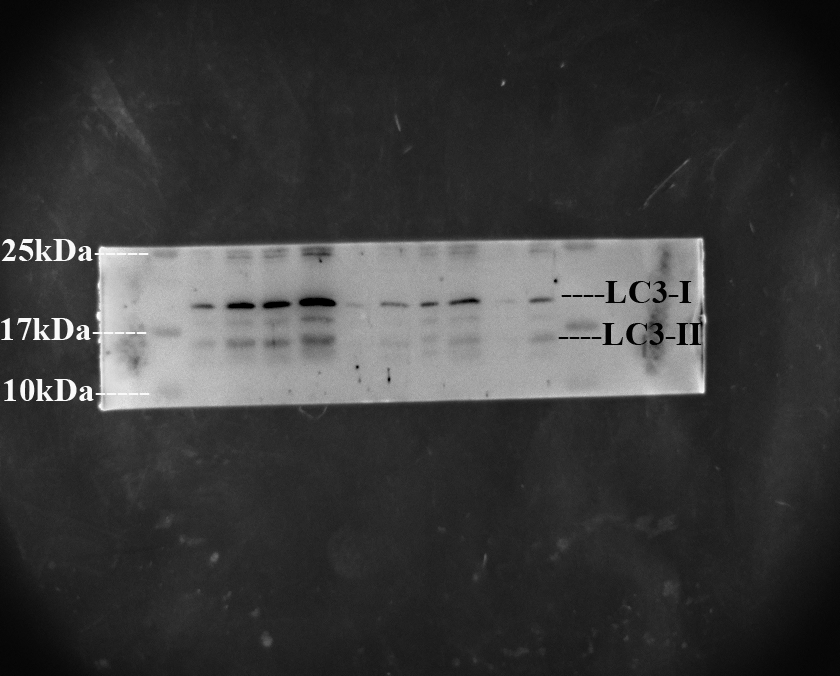


p62


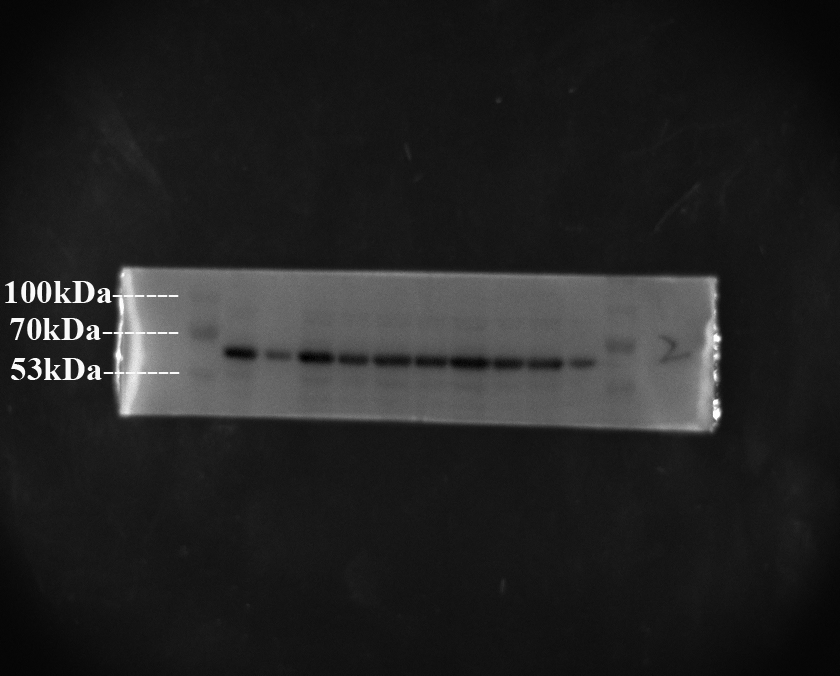


LC3II


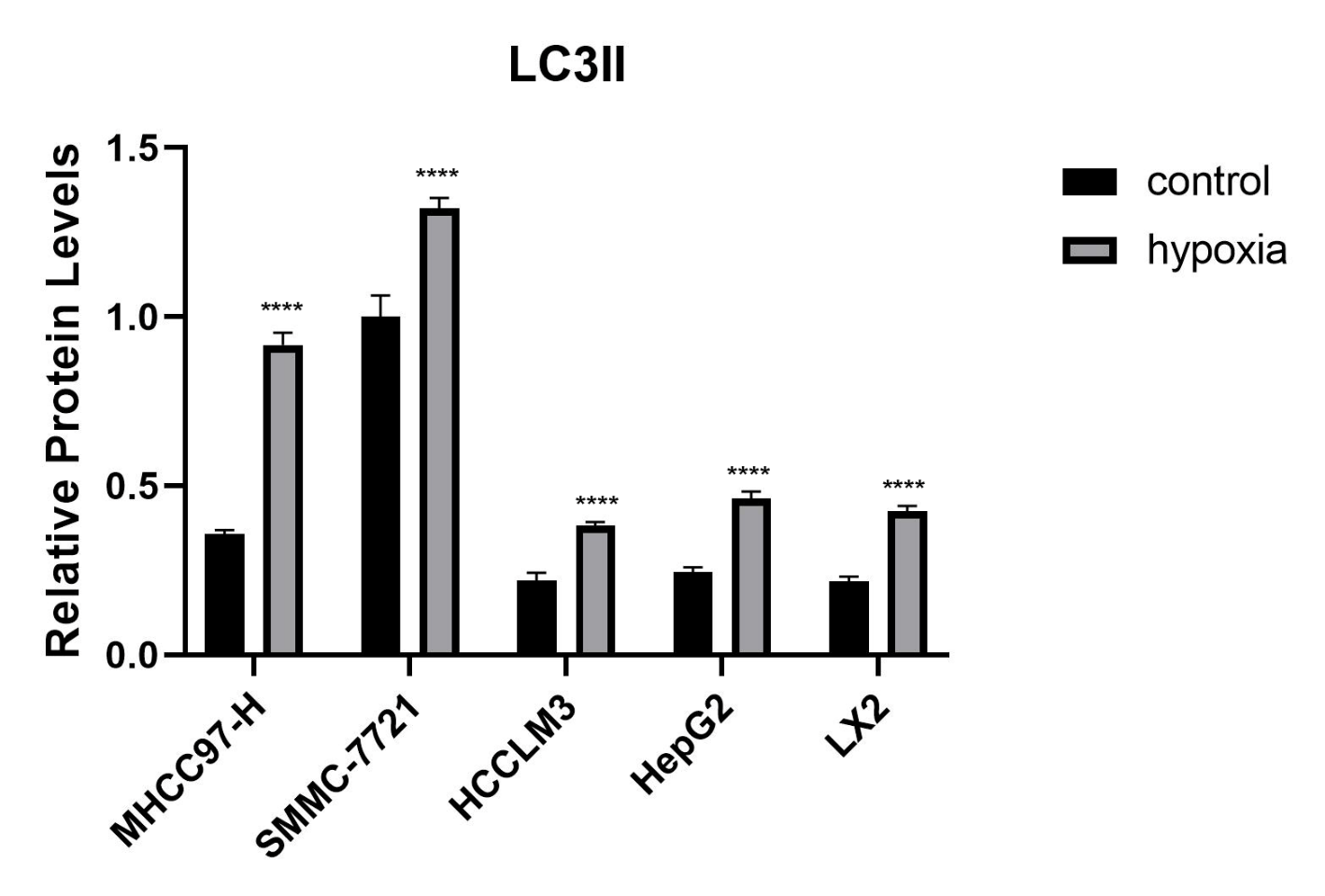


p62


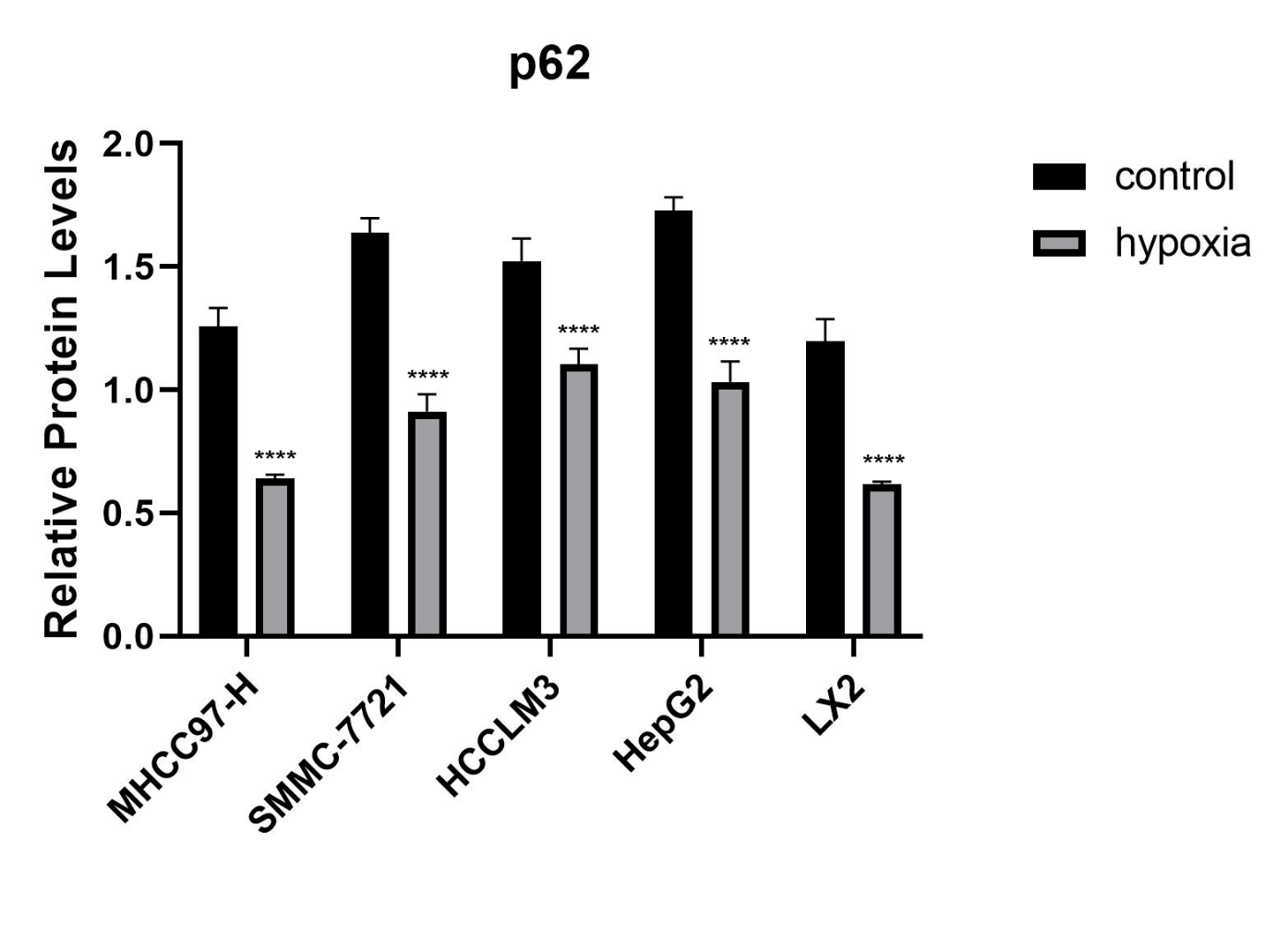

Supplement: Supplementary file 1 — Additional file1:. Figure S1. The original images of western blot. [file 12863_2022_1055_MOESM1_ESM.docx]
